# The combined effects of cholesteryl ester transfer protein (*CETP*) TaqIB gene polymorphism and canola, sesame and sesame-canola oils consumption on metabolic response in patients with diabetes and healthy people

**DOI:** 10.34172/jcvtr.2020.32

**Published:** 2020-08-02

**Authors:** Nahid Ramezani-Jolfaie, Shiva Aghaei, Ehsan Farashahi Yazd, Ali Moradi, Hassan Mozaffari-Khosravi, Mojgan Amiri, Hamidreza Raeisi-Dehkordi, Fatemeh Moghtaderi, Alireza Zimorovat, Seyed Ali Yasini Ardakani, Amin Salehi-Abargouei

**Affiliations:** ^1^Nutrition and Food Security Research Center, Shahid Sadoughi University of Medical Sciences, Yazd, Iran; ^2^Department of Nutrition, School of Public Health, Shahid Sadoughi University of Medical Sciences, Yazd, Iran; ^3^Stem Cell Biology Research Center, Yazd Reproductive Sciences Institute, Shahid Sadoughi University of Medical Sciences, Yazd, Iran; ^4^Department of Biochemistry and Molecular Biology, Faculty of Medicine, Shahid Sadoughi University of Medical Sciences, Yazd, Iran; ^5^Yazd Diabetic Research Center, Shahid Sadoughi University of Medical Sciences, Yazd, Iran; ^6^Department of Food Science and Technology, Islamic Azad University, Yazd, Iran

**Keywords:** Cholesterol Ester Transfer Proteins, Diabetes Mellitus, Sesame Oil, Canola Oil, Sesame-Canola Oil, Polymorphism

## Abstract

***Introduction:*** Cholesteryl ester transfer protein (CETP) is a key regulating enzyme in the lipid metabolism pathway, and its gene polymorphism may be a candidate for modulating the metabolic responses to dietary intervention. We thus examined whether the effects of the CETP TaqIB polymorphism on metabolic profiles were modified by dietary plant oils.

***Methods:*** This is a retrospective analysis of data collected during a randomized triple-blind cross over trial. A total of 95 patients with type 2 diabetes and 73 non-diabetes individuals completed a 9-weekof the intake of sesame, canola and sesame-canola oils. Blood samples were collected at the beginning and at the end of each intervention period for biochemical analysis. Genotyping was done using the polymerase chain reaction-restriction fragment length polymorphism (PCR-RFLP) method.

***Results:*** In diabetes patients, B1B1 homozygotes of the CETP TaqIB polymorphism compared with B2 carriers (B1B2 + B2B2) had significantly lower diastolic blood pressure, apoB and apoB: apoA-1,and higher Lp(a) after the intake of sesame-canola oil, as well as lower insulin and HOMA-IR after the intake of sesame oil. There was also a significant effect of genotype on adjusted changes of apoB, apoB: apoA-1, insulin, HOMA-IR and QUICKI. A significant genotype-dietary oils combined effects were observed for diastolic blood pressure, and LDL: HDL, TC: HDL and TG: HDL ratios in diabetes patients. No independent or combined effects of dietary oils and genotypes on outcomes were found in healthy people.

***Conclusion:*** There was a modulatory effect of the CETP TaqIB polymorphism on some metabolic traits in response to plant oils in patients with diabetes. Taken together, the intake of sesame-canola and canola oils showed more favorable effects in diabetes patients with B1B1 genotype. Future investigations are needed to confirm these results.

## Introduction

The effects of changes in dietary fat quality on lipid and glycemic metabolism have been extensively studied. A large body of evidence has suggested that a reduction of saturated fatty acids (SFAs) and its substitution with unsaturated fatty acids (UFAs) might help to improve insulin sensitivity and circulating lipid levels, as well as cardiovascular risk.^1-4^ Some of plant-based oils that contain considerable amounts of mono- (MUFAs) and polyunsaturated fatty acids (PUFAs) are being investigated as suitable alternatives for dietary oils rich in saturated and trans-fatty acids.^5^ Healthy vegetable oils such as canola and sesame oils with high contents of MUFAs (e.g. oleic acid), PUFAs (e.g. alpha-linolenic acid and linoleic acid), and antioxidants (e.g. tocopherol, lignans, and phytosterols) have been reported to be beneficial in improving cardiovascular risk factors, although some other studies were controversial.^6-10^ These inconsistent observations have been proposed to be related to the combined effects of genetic and environmental factors (e.g. dietary intake) that justified different individual responses to dietary interventions in general.^11^ Genetic variations including single-nucleotide polymorphisms (SNPs) in the genes that encode proteins involved in lipid and glycemic metabolism, may play a major role in changes in metabolic profile.^12^

The human*cholesteryl ester transfer protein (CETP)* gene is localized on chromosome 16 in the region of q21 (16q21) and contained 16 exons and 15 introns.^13^ This gene encodes a protein containing 476 amino acids as one of the key enzymes in lipid metabolism that plays an important role in reverse-cholesterol transport resulting in decreasing high-density lipoprotein (HDL) cholesterol and increasing the cholesterol content of low-density lipoprotein (LDL) and very low-density lipoprotein (VLDL) cholesterol. Indeed, it transfers cholesterol ester from HDL to apo B-containing lipoproteins such as LDL, VLDL and VLDL remnants in exchange for triglyceride and subsequent uptake of cholesterol by hepatocytes.^14^ TaqIB is a common polymorphism in the *CETP* gene, which is located in nucleotide 277 of intron 1 and characterized by a silent base change from G [called as B1 allele (presence of the TaqI endonuclease restriction site) (frequent allele)] to A [called as B2 allele (absence of the TaqI endonuclease restriction site) (less common allele)].^15^

Meta-analyses have associated the TaqIB polymorphism with HDL-C and coronary diseases, such that individuals carrying the B2 allele are reported to have higher HDL-C levels and lower risk of coronary diseases than B1B1 homozygotes.^16,17^ The presence of the B2 allele has also been associated with moderate inhibition of CETP activity.^17^ Furthermore, another research provided evidence regarding the effect of this polymorphism on parameters of insulin resistance.^18^ In previous studies that evaluated the combined effects of the TaqIB polymorphism and dietary intakes, a lack of consistency was observed. A number of researchers suggested a potential interaction between this polymorphism and type and amount of dietary fat on serum lipid and lipoprotein levels.^19-23^ However, the findings of other studies did not support that the TaqIB polymorphism can affect metabolic responses to dietary intakes.^24-27^

Although there are several studies regarding the combined effects of *CETP* polymorphisms and different dietary interventions, we found no study that assessed the potential combined effects of *CETP* genotypes and plant oils on the determination of cardiometabolic profile. Therefore, we decided to discover whether the effects of sesame, canola, and sesame-canola oils on blood lipids and glycemic control markers are conditioned by the *CETP* TaqIB polymorphism in patients with type 2 diabetes and healthy people.

## Materials and Methods

### Participants

This study was conducted within a trial that was registered in the Iranian Registry of Clinical Trials (identifier: IRCT2016091312571N6) and ethically approved by the ethics committee of Shahid Sadoughi University of Medical Sciences, Yazd, Iran (IR.SSU.SPH.REC.1397.139). All participants provided written informed consent. A detailed description of the methodology of the trial has been published, elsewhere.^28^ In brief, patients with type 2 diabetes and their spouses were recruited from the general population referred to the Yazd Diabetes Research Center of Shahid Sadoughi University of Medical Sciences. Patients with type 2 diabetes had following criteria; (1) history of diabetes for at least 6 months or maximum 10 years (fasting blood glucose more than 126 mg/dL and/or HbA1c more than 6.5% and less than 8%),^29^ (2) being on oral anti-glycemic medications without insulin therapy, (3) no change in dosage of lipid-lowering drugs at least for 3 months before beginning the study. Those healthy spouses who were without diabetes (fasting blood glucose less than 126 mg/dL and/or HbA1c less than 6.5%), were also included. All of the participants aged more than 18 years old and had no history of any diseases such as cardiovascular disorders (coronary artery disease, stroke, congestive heart disease) and coronary artery bypass grafting, renal diseases, liver disorders (serum glutamic oxaloacetic transaminase and serum glutamic pyruvic transaminase three times more than normal values), and cancers. Subjects were excluded from the study if they changed dietary food habits considerably, went on insulin therapy, get pregnant, get chronic diseases, and decided to discontinue the study due to any reason.^28^

### Sampling size

This is a retrospective analysis of data collected during the parent study. A sample size of n = 34 in total was calculated based on a formula suggested for cross-over studies as follows^30^: n = [(z 1−α/2+z 1−β)^2 *^ s^2^]/2Δ^2^; assuming the type one error of 5% and the type 2 error of 10% (power of 90%) and serum glucose as the key variable.^31^ Since the investigators aimed to perform sex specific analyses in the parent study, and also with considering the probability of high rate of attrition, 50 men and 50 women with type 2 diabetes and their healthy spouses who had eligibility criteria were recruited in the parent study.

### Procedure

The study involved a randomized triple-blind crossover trial of three interventions: 1) sesame oil, 2) canola oil, and 3) sesame-canola oil (40% sesame oil and 60% canola oil). Participants at first followed a run-in period of 4 weeks during which regular oils used in households were substituted with sunflower oil. Thereafter, they went through three 9-week intervention periods which separated by 4-week washout periods. The participants were asked to consume sunflower oil for 4 weeks in washout periods. The oil packs were similar in shape and labeled with three codes (S, B, and G) by a person who was not informed about the study protocol. All of the intervention oils were provided by Neshatavar food industry (Datis Corporation) free of charge for all family members of the participants and were fully replaced with any edible oils that the subjects regularly used at their homes. Indeed, the participants were not allowed to consume any other edible oil during the study period. The amount of consumed oils that estimated by using dietary records and weighing the given and returned oil bottles, was approximately 31 g per individual. Gas chromatography with flame ionized detector (GC-FID) was also used to detect the fatty acid composition of the intervention oils. The fatty acids profile has been provided, previously.^28^

### Measurements

All assessments in all phases of the research were completed by trained researchers blinded to the treatment protocol.

#### Dietary intake and physical activity

At the beginning, middle, and end of each treatment period, a 3-day weighed food record and a 3-day physical activity record (2 weekdays and 1 weekend day) were obtained from the participants. All participants were requested to follow their usual physical activity and dietary habits during the study.

#### Anthropometric and blood pressure measurements

The participants attended the clinic at the beginning, middle, and end of each treatment period for measuring anthropometric parameters and blood pressure. Weight was recorded with an accuracy of 100 g, using a digital calibrated scale (Omron, Japan, model: BF51) with light clothes and no shoes. Height was recorded with an accuracy of 0.1 cm, using a wall-fixed measuring tape, in standing position with shoulders in normal alignment and no shoes. Body mass index (BMI) was calculated as body weight (kg) divided by height squared (m^2^). Systolic/diastolic blood pressure (SBP/DBP) was measured after a 5-minute rest in a sitting position, using a barometer (Riester, model: Diplomat-presameter). Three measures were taken and averaged for all variables.

#### Laboratory

At the beginning and at the end of each intervention period, the venous blood samples were drawn after a 10-12 h overnight fast and stored at -80ºC until analyzed. The biochemical analyses of blood lipids, lipoproteins, apolipoproteins, fasting blood glucose (FBG) and liver enzymes were conducted by an auto-analyzer (Alpha-classic, model: AT++) using Pars Azmun kits (Pars Azmun Co., Iran).Fasting serum insulin concentrations were measured using enzyme-linked immunoassay (ELISA) kits (Monobind, Inc., Lake Forest, CA, USA). The quantitative insulin sensitivity check index (QIUCKI) and hemostatic model assessment of insulin resistance (HOMA-IR) were calculated with the use of suggested formulas.^32,33^

### DNA extraction and genotyping

**Figure 1:**
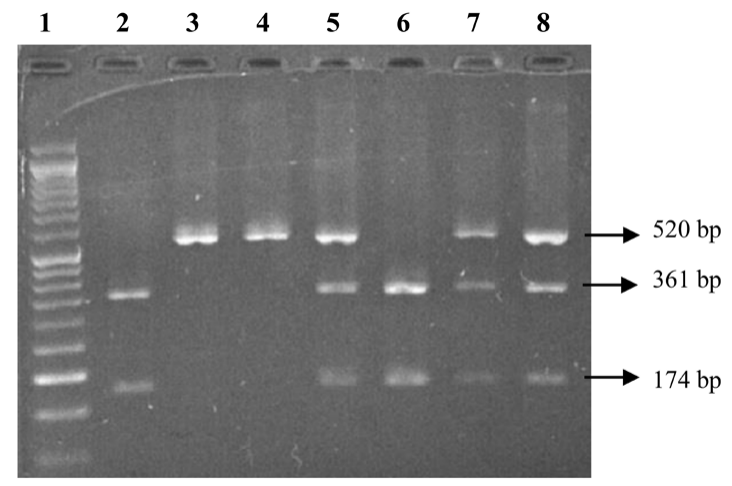
The PCR-RFLP electrophoresis of the TaqIB *CETP* polymorphism on 2% agarose gel.50 bp ladder marker (Lane 1); 361 bp and 174 bp showing homozygous B1B1 genotype (lane 2 and 6); 520 bp, 361 bp, and 174 bp showing heterozygous B1B2 genotype (lane 5, 7, and 8); 520 bp showing homozygous B2B2 genotype (lane 3 and 4)

Genomic DNA was extracted from 250 µL of whole blood using the DNJia Blood Kit (Roje Technologies Inc, Iran) based on silica technology. The TaqIB SNP (rs708272) was determined by amplifying a fragment of 520 base pairs (bp) in intron 1 of the *CETP* gene by polymerase chain reaction-restriction fragment length polymorphism (PCR-RFLP) method. The PCR mixture was provided in a total volume of 20 µL containing 1 µL of genomic DNA, 10 µL of Master Mix (Ampliqon, Denmark), 8 µL of water and 0.5 µL (5 pmol) of each oligonucleotide primer (forward: 5’-ACTAGCCCAGAGAGAGGAGTG-3’ and reverse: 5’-CAGCCGCACACTAACCCTA-3’). DNA was denatured at 95°C for 5 minutes; this was followed by 40 cycles of amplification at 95°C for 30 seconds, annealing at 66°C for 30 seconds, extension at 72°C for 30 seconds, and ended with a final extension at 72°C for 5 minutes. The PCR products were digested with 5 units of the restriction endonuclease enzyme TaqI (Fermentase, Lithuania) in a total volume of 20 µL after incubation at 37°C overnight. The digested DNA fragments (8 µL) were loaded on 2% agarose gel (SinaClon, Iran) and subjected to electrophoresis for 1.5 hours at 100 V, and were finally visualized by an ultraviolet transilluminator. The B1 allele has two bands of 361 and 174 bp, and the B2 allele is characterized by one fragment of 520 bp in length. The PCR-RFLP electrophoresis of the TaqIB *CETP* polymorphism on 2% agarose gel is provided in Figure 1. The accuracy of the genotyping was confirmed using direct gene sequencing of randomly selected samples.

### Statistical analysis

All data presented in the text and tables are expressed as mean ± standard error (SE). Statistical analyses were performed with the SPSS package, version 24.0 (IBM Corporation, USA). Statistical significance was defined as *P* values < 0.05. All analyses were separately performed in diabetes and healthy people. Normal distribution for all continuous variables was assessed by graphical methods and hypotheses tests. One-way analysis of variance (ANOVA) was used to multiple comparisons of baseline means between three genotypes (B1B1, B1B2, and B2B2). A mixed linear model followed by Bonferroni post hoc comparisons was used to determine the changes of mean values (with SEs) from baseline to end of treatment periods, disaggregated by genotype. Within-period comparisons were also performed using a mixed linear model. Models were adjusted for age, gender, baseline BMI, amount of consumed oils, change levels of physical activity, and change in energy intake. The gene-diet interaction was tested to determine whether the observed effects in each intervention period are dependent on the *CETP* TaqIB genotype.

## Results

**Figure 2:**
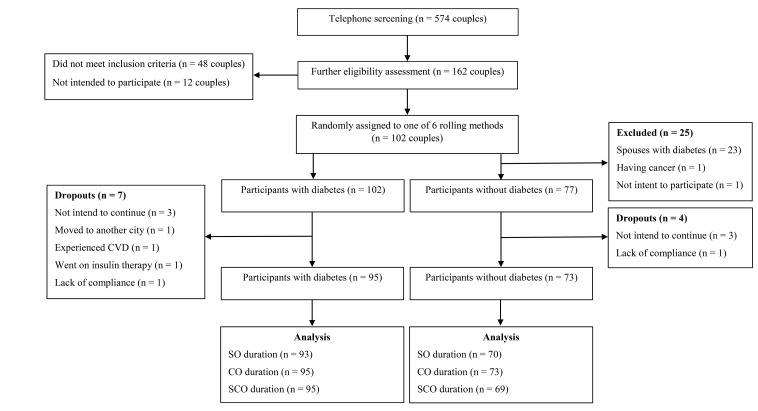
The flow diagram of the attendance of study participants. CVD, cardiovascular disease; CO, canola oil; SO, sesame oil; SCO, sesame-canola oil

In total, 95 patients with type 2 diabetes (49 females; 46 males) and 73 healthy people (41 females; 32 males) completed the study protocol. The flow diagram of the attendance of study participants is provided in Figure 2. Genotype frequency of the *CETP* TaqIB polymorphism was as follows: 18.9% for B1B1, 61.1% for B1B2 and 20% for B2B2 in patients with diabetes; 13.7% for B1B1, 64.4% for B1B2 and 21.9% for B2B2 in healthy people. The baseline characteristics of the participants according to the *CETP* TaqIB genotypes are shown in Table S1. All variables did not differ statistically between genotypes in both type 2 diabetes and healthy people.

Total energy and energy percent of protein, carbohydrate, and fat intake as well as physical activity had no significant differences between the intervention periods in subjects with and without diabetes. The mean intake of MUFAs and PUFAs in diabetes patients and also SFAs in healthy people considerably differ between the intervention periods (Table S2).

### The effect of dietary oils on anthropometric measures and blood pressure according to the CETP TaqIB genotypes

As shown in Tables 1 and 2, in both patients with type 2 diabetes and healthy people, there was no significant change in outcomes based on the *CETP* TaqIB genotypes for all three treatment periods (*P* > 0.05). No independent or combined effects of dietary oils and genotypes were also found. However, in patients with type 2 diabetes, a significant difference for adjusted DBP changes was observed between B1B2 and B2B2 genotypes during the sesame-canola oil period (-0.23 ± 0.15 mm Hg vs. 0.79 ± 0.27 mm Hg). In addition, a significant combined effect of dietary oils and genotypes was observed (*P* = 0.039, Table 1). Specifically, B1B2 genotype group showed a decreased change in DBP following the intake of sesame-canola oil, but an increase in DBP was observed in sesame and canola oil periods.

**Table 1:** Change values in anthropometric measures, blood pressure, lipoproteins, apolipoproteins, and glycemic indices in patients with type 2 diabetes across treatment periods according to the *CETP* TaqIB genotypes^1^

	**Sesame oil**			**Sesame-Canola oil**			**Canola oil**			***P**^1^*	***P**^2^*	***P**^3^*
	**B1B1**	**B1B2**	**B2B2**	**B1B1**	**B1B2**	**B2B2**	**B1B1**	**B1B2**	**B2B2**			
Weight, kg	0.27 (0.36)	0.22 (0.20)	0.68 (0.36)	-0.32 (0.34)	0.06 (0.19)	0.02 (0.35)	0.25 (0.37)	0.27 (0.21)	0.62 (0.38)	0.140	0.245	0.885
BMI, kg/m^2^	0.10 (0.13)	0.08 (0.07)	0.25 (0.13)	-0.11 (0.13)	0.01 (0.07)	0.01 (0.13)	0.08 (0.13)	0.09 (0.07)	0.21 (0.14)	0.160	0.285	0.914
WC, cm	-0.67 (0.43)	-0.88 (0.24)	0.05 (0.43)	-0.83 (0.59)	-0.59 (0.34)	-1.08 (0.61)	-0.08 (0.56)	-0.81 (0.32)	-0.47 (0.58)	0.629	0.560	0.507
Visceral fat, %	0.18 (0.14)	0.08 (0.08)	0.21 (0.14)	-0.17 (0.28)	0.10 (0.15)	-0.63 (0.28)	0.14 (0.12)	0.12 (0.07)	0.17 (0.13)	0.052	0.263	0.216
Body fat, %	0.12 (0.51)	0.29 (0.28)	0.17 (0.50)	-0.05 (0.43)	-0.05 (0.24)	0.78 (0.43)	0.02 (0.32)	0.37 (0.18)	0.43 (0.33)	0.956	0.362	0.728
Muscle mass, %	-0.04 (0.20)	0.00 (0.10)	-0.21 (0.19)	0.04 (0.21)	-0.002 (0.11)	-0.16 (0.21)	0.03 (0.19)	-0.23 (0.11)	-0.27 (0.19)	0.741	0.258	0.925
SBP, mm Hg	0.26 (0.30)	0.27 (0.17)	-0.49 (0.30)	0.33 (0.30)	-0.30 (0.17)	0.05 (0.30)	0.19 (0.27)	0.06 (0.15)	-0.21 (0.27)	0.998	0.086	0.219
DBP, mm Hg	0.14 (0.27)	0.43 (0.16)	-0.02 (0.27)	0.11 (0.27)	-0.23 (0.15)^a^	0.79 (0.27)^b^	0.17 (0.26)	0.25 (0.15)	0.20 (0.27)	0.983	0.467	**0.039**
TC, mg/dL	1.76 (6.55)	1.81 (3.71)	3.35 (6.55)	-10.22 (7.00)	1.38 (4.00)	2.79 (7.20)	3.75 (6.06)	3.17 (3.47)	-1.50 (6.24)	0.627	0.730	0.624
HDL-C, mg/dL	-0.27 (2.14)	0.25 (1.17)	-1.02 (2.07)	2.18 (1.77)	0.04 (1.01)	0.08 (1.83)	0.51 (1.80)	2.19 (1.04)	0.11 (1.85)	0.665	0.656	0.668
LDL-C, mg/dL	-1.55 (3.72)	0.68 (2.10)	1.50 (3.72)	-5.63 (4.22)	0.06 (2.41)	0.88 (4.34)	-0.13 (3.68)	1.69 (2.11)	-0.38 (3.79)	0.757	0.497	0.917
TG, mg/dL	30.47 (18.57)	4.25 (10.52)	1.20 (18.57)	-33.63 (15.98)	9.73 (9.14)	0.55 (16.44)	7.69 (16.31)	-8.00 (9.33)	6.70 (16.78)	0.314	0.992	0.064
Lp(a), mg/dL	-2.99 (3.20)	0.63 (1.77)	-1.35 (3.01)	6.37 (2.17)^a^	0.20 (1.16)^b^	-0.59 (2.17)^b^	-3.13 (3.58)	-0.07 (1.98)	-3.63 (3.83)	0.054	0.590	0.107
LDL: HDL	-0.12 (0.17)	0.01 (0.09)	0.13 (0.17)	-1.29 (0.50)	-0.03 (0.28)	0.06 (0.51)	0.75 (0.44)	-0.27 (0.25)	-0.04 (0.45)	0.161	0.762	**0.027**
TC: HDL	-0.20 (0.36)	0.05 (0.20)	0.25 (0.35)	-2.82 (1.11)	-0.01 (0.64)	0.17 (01.15)	1.67 (0.94)	-0.61 (0.54)	-0.11 (0.96)	0.179	0.793	**0.024**
TG: HDL	-0.23 (0.87)	0.21 (0.47)	0.19 (0.84)	-7.00 (2.81)	0.17 (1.61)	0.20 (2.89)	3.69 (2.15)	-1.66 (1.24)	0.10 (2.21)	0.192	0.733	**0.025**
ApoB, mg/dL	-5.14 (4.87)	-0.03 (2.75)	2.08 (4.87)	-19.05 (6.12)^a^	-0.09 (3.50)^b^	2.79 (6.29)^b^	0.97 (5.20)	0.59 (2.97)	-0.20 (5.35)	0.374	**0.041**	0.284
ApoA-1, mg/dL	-0.52 (4.54)	-1.37 (2.57)	-0.47 (4.54)	-4.22 (4.625)	0.14 (2.64)	-1.02 (4.75)	11.22 (5.20)	5.06 (2.97)	-1.82 (5.35)	0.143	0.709	0.474
ApoB: ApoA-1	-0.03 (0.03)	0.008 (0.02)	0.02 (0.03)	-0.12 (0.04)^a^	-0.006 (0.02)^b^	0.01 (0.04)^b^	-0.02 (0.03)	-0.02 (0.01)	0.004 (0.03)	0.501	**0.018**	0.585
FBG, mg/dL	0.76 (4.74)	0.34 (2.68)	5.85 (4.74)	1.50 (7.27)	-4.84 (4.15)	0.32 (7.48)	2.75 (6.92)	9.00 (3.96)	9.17 (7.12)	0.254	0.740	0.821
Insulin, mIU/mL	-15.07 (3.77)^a^	-4.75 (2.20)^b^	0.76 (3.77)^b^	-2.23 (3.48)	-6.36 (1.99)	-3.60 (3.58)	-6.97 (3.45)	-2.61 (1.88)	1.46 (3.57)	0.411	**0.031**	0.086
HOMA-IR	-1.74 (0.45)^a^	-0.61 (0.26)	0.10 (0.45)^b^	-0.28 (0.41)	-0.78 (0.23)	-0.48 (0.43)	-0.88 (0.44)	-0.27 (0.24)	0.35 (0.44)	0.379	**0.024**	0.082
QUICKI	0.015 (0.006)	0.009 (0.003)	0.000 (0.006)	0.006 (0.006)	0.011 (0.003)	0.008 (0.006)	0.015 (0.006)	0.001 (0.003)	-0.005 (0.006)	0.437	**0.046**	0.245
ALP, U/L	0.76 (5.96)	-0.64 (3.38)	-4.02 (5.96)	-1.58 (7.42)	0.68 (4.24)	-16.52 (7.63)	-3.25 (4.88)	3.92 (2.79)	6.23 (5.03)	0.200	0.319	0.272
GGT, U/L	1.31 (2.68)	-1.47 (1.52)	-0.43 (2.68)	-0.35 (2.95)	1.09 (1.69)	1.52 (3.04)	2.44 (1.87)	1.02 (1.07)	3.04 (1.92)	0.275	0.586	0.928
AST, U/L	2.40 (1.72)	1.00 (0.97)	-2.49 (1.72)	-4.24 (1.94)	-0.80 (1.11)	-0.53 (2.00)	2.24 (2.08)	-1.55 (1.19)	-0.97 (2.15)	0.279	0.656	0.102
ALT, U/L	1.97 (2.41)	0.01 (1.36)	-0.73 (2.41)	-2.87 (2.28)	-1.56 (1.30)	-1.60 (2.35)	3.81 (3.08)	-1.57 (1.76)	0.89 (3.17)	0.183	0.537	0.607

^1^ All data are presented as mean ± standard error. ApoA-1, apolipoprotein A-1; ApoB, apolipoprotein B; ALP, alkaline phosphatase; AST, aspartate aminotransferase; ALT, alanine aminotransferase, BMI, body mass index; DBP, diastolic blood pressure; FBG, fasting blood glucose; GGT, gamma-glutamyltransferase; HDL-C, High-density lipoprotein cholesterol; HOMA-IR, homeostasis model assessment for insulin resistance; LDL-C, Low-density lipoprotein cholesterol; Lp(a), lipoprotein a; QUICKI, quantitative insulin sensitivity check index; SBP, Systolic blood pressure; TC, total cholesterol; TG, Triglyceride; WC, waist circumference. ^a,b^ values with different superscripts are statistically significant (within treatment period comparisons of change values between genotypes using linear mixed models, adjusted for age, gender, baseline BMI, amount of consumed oils, change levels of physical activity and change in energy intake). *P* ^1^, comparisons of change values between the treatment oils using linear mixed models, adjusted for age, gender, baseline BMI, amount of consumed oils, change levels of physical activity and change in energy intake. *P* ^2^, comparisons of change values between the genotypes using linear mixed models, adjusted for age, gender, baseline BMI, amount of consumed oils, change levels of physical activity and change in energy intake. *P* ^3^, interaction between TaqIB SNP and treatment oils on the outcomes of interest, adjusted for age, gender, baseline BMI, amount of consumed oils, change levels of physical activity and change in energy intake.

**Table 2:** Change values in anthropometric measures, blood pressure, lipoproteins, apolipoproteins, and glycemic indices in healthy people across treatment periods according to the *CETP* TaqIB genotypes^1^

	**Sesame oil**			**Sesame-Canola oil**			**Canola oil**			***P**^1^*	***P**^2^*	***P**^3^*
	**B1B1**	**B1B2**	**B2B2**	**B1B1**	**B1B2**	**B2B2**	**B1B1**	**B1B2**	**B2B2**			
Weight, kg	0.75 (0.47)	-0.05 (0.21)	0.02 (0.36)	0.06 (1.32)	0.01 (0.60)	-1.71 (0.99)	0.26 (0.41)	0.00 (0.19)	0.35 (0.33)	0.441	0.421	0.403
BMI, kg/m^2^	0.27 (0.17)	-0.02 (0.08)	0.01 (0.13)	0.00 (0.54)	-0.001 (0.24)	-0.71 (0.40)	0.09 (0.15)	0.00 (0.07)	0.13 (0.12)	0.419	0.421	0.404
WC, cm	-0.44 (0.66)	-1.10 (0.30)	-0.40 (0.51)	-0.66 (0.75)	-0.61 (0.34)	-1.46 (0.56)	-1.11 (0.80)	-0.46 (0.36)	-0.17 (0.64)	0.779	0.990	0.435
Visceral fat, %	0.22 (0.52)	-0.17 (0.24)	-0.20 (0.40)	-0.22 (0.19)	0.14 (0.09)	0.00 (0.14)	0.00 (0.16)	0.00 (0.07)	-0.14 (0.13)	0.980	0.822	0.654
Body fat, %	0.37 (0.45)	0.15 (0.20)	-0.09 (0.34)	-0.10 (0.48)	0.29 (0.22)	0.23 (0.36)	0.40 (0.38)	0.37 (0.17)	0.28 (0.31)	0.719	0.805	0.911
Muscle mass, %	-0.20 (0.26)	-0.08 (0.12)	0.13 (0.20)	0.03 (0.30)	-0.21 (0.14)	-0.09 (0.22)	-0.27 (0.48)	-0.27 (0.22)	-0.85 (0.39)	0.262	0.878	0.534
SBP, mm Hg	-0.33 (1.60)	0.14 (0.73)	-2.10 (1.23)	-0.22 (0.43)	-0.18 (0.19)	-0.09 (0.32)	-0.05 (0.41)	0.00 (0.19)	0.25 (0.33)	0.420	0.424	0.571
DBP, mm Hg	-0.11 (0.38)	-0.09 (0.17)	0.30 (0.29)	0.33 (0.36)	0.00 (0.16)	-0.06 (0.27)	-0.27 (0.34)	0.10 (0.15)	0.21 (0.27)	0.938	0.702	0.633
TC, mg/dL	1.88 (7.52)	-2.75 (3.52)^a^	17.06 (5.82)^b^	-0.66 (9.23)	-0.36 (4.17)	-2.00 (7.15)	-12.88 (7.39)	1.50 (3.42)	1.42 (5.92)	0.156	0.242	0.103
HDL-C, mg/dL	0.83 (2.90)	1.84 (1.36)	1.50 (2.24)	-1.16 (2.54)	-1.75 (1.15)	1.56 (1.96)	1.27 (3.20)	0.20 (1.48)	1.10 (2.56)	0.517	0.680	0.857
LDL-C, mg/dL	0.72 (4.89)	-1.68 (2.29)	8.30 (3.79)	0.38 (5.78)	-0.95 (2.61)	-1.56 (4.48)	-10.11 (4.48)	0.96 (2.07)	0.39 (3.59)	0.136	0.352	0.092
TG, mg/dL	-4.22 (21.46)	-11.68 (10.05)	-7.90 (16.62)	-15.22 (16.16)	15.44 (7.31)	-6.03 (12.52)	-8.66 (22.14)	11.53 (10.25)	-2.03 (17.75)	0.858	0.323	0.748
Lp(a), mg/dL	1.30 (4.20)	0.92 (1.87)	5.08 (3.29)	-0.15 (4.86)	0.22 (2.09)	4.51 (3.67)	-1.49 (4.25)	0.83 (1.88)	-2.29 (3.33)	0.541	0.764	0.805
LDL: HDL	-0.08 (0.57)	-0.32 (0.27)	0.11 (0.44)	0.01 (0.24)	-0.005 (0.10)	0.01 (0.187)	-0.29 (0.26)	-0.06 (0.12)	-0.01 (0.21)	0.641	0.713	0.879
TC: HDL	-0.14 (1.26)	-0.70 (0.59)	0.23 (0.98)	-0.02 (0.47)	0.02 (0.21)	0.02 (0.36)	-0.38 (0.46)	-0.17 (0.21)	-0.03 (0.37)	0.720	0.697	0.931
TG: HDL	-0.33 (2.97)	-1.42 (1.39)	-0.31 (2.30)	-0.58 (1.03)	0.14 (0.46)	-0.04 (0.79)	-0.19 (0.94)	-0.01 (0.44)	-0.04 (0.75)	0.891	0.908	0.959
ApoB, mg/dL	4.44 (8.13)	-4.12 (3.81)	6.73 (6.30)	-4.83 (8.28)	-0.42 (3.74)	1.66 (6.41)	-11.55 (7.06)	-0.02 (3.27)	5.10 (5.66)	0.614	0.259	0.484
ApoA-1, mg/dL	-2.11 (7.36)	-3.63 (3.45)	4.60 (5.70)	-8.22 (8.18)	-2.53 (3.70)	4.66 (6.33)	8.44 (7.78)	6.57 (3.60)	-5.42 (6.24)	0.404	0.962	0.102
ApoB: ApoA-1	0.05 (0.06)	-0.01 (0.02)	0.03 (0.04)	0.009 (0.08)	0.03 (0.03)	0.001 (0.06)	-0.12 (0.05)	-0.02 (0.02)	0.04 (0.04)	0.259	0.677	0.287
FBG, mg/dL	7.22 (4.74)	-0.98 (2.22)	3.53 (3.67)	-3.05 (3.14)	1.62 (1.42)	3.43 (2.43)	-1.00 (3.93)	5.97 (1.82)	1.03 (3.15)	0.624	0.819	0.097
Insulin, mIU/mL	-3.65 (3.80)	-3.31 (1.87)	-7.08 (2.95)	0.74 (4.38)	-0.26 (2.00)	-3.59 (3.39)	-5.96 (5.43)	-3.96 (2.54)	-6.27 (4.36)	0.270	0.501	0.981
HOMA-IR	-0.37 (0.45)	-0.40 (0.22)	-0.80 (0.35)	0.05 (0.49)	-0.05 (0.22)	-0.42 (0.38)	-0.70 (0.61)	-0.44 (0.28)	-0.69 (0.49)	0.327	0.550	0.980
QUICKI	0.008 (0.007)	0.008 (0.003)	0.010 (0.005)	0.000 (0.008)	0.003 (0.004)	0.010 (0.006)	0.014 (0.009)	0.006 (0.004)	0.010 (0.007)	0.573	0.676	0.927
ALP, U/L	10.77 (8.04)	-5.68 (3.77)	4.93 (6.23)	-6.22 (8.01)	1.63 (3.62)	0.33 (6.21)	-3.00 (6.86)	2.00 (3.17)	9.78 (5.50)	0.619	0.306	0.272
GGT, U/L	-1.83 (4.53)	-3.17 (2.12)	-1.33 (3.51)	0.31 (2.24)	-0.70 (1.01)	-0.92 (1.73)	-1.23 (3.15)	3.50 (1.46)	4.12 (2.53)	0.316	0.774	0.715
AST, U/L	-0.73 (2.27)	0.88 (1.06)	-1.18 (1.76)	2.94 (3.06)	-3.85 (1.38)	1.68 (2.37)	-4.27 (1.87)^a^	-0.93 (0.87)^b^	3.02 (1.50)^b^	0.828	0.145	**0.019**
ALT, U/L	-2.54 (2.96)	-0.52 (1.38)	-3.40 (2.29)	0.53 (3.98)	-4.39 (1.80)	0.48 (3.08)	-2.97 (3.41)^a^	1.66 (1.58)	7.70 (2.73)^b^	0.122	0.286	0.080

^1^All data are presented as mean ± standard error. ApoA-1, apolipoprotein A-1; ApoB, apolipoprotein B; ALP, alkaline phosphatase; AST, aspartate aminotransferase; ALT, alanine aminotransferase, BMI, body mass index; DBP, diastolic blood pressure; FBG, fasting blood glucose; GGT, gamma-glutamyltransferase; HDL-C, High-density lipoprotein cholesterol; HOMA-IR, homeostasis model assessment for insulin resistance; LDL-C, Low-density lipoprotein cholesterol; Lp(a), lipoprotein a; QUICKI, quantitative insulin sensitivity check index; SBP, Systolic blood pressure; TC, total cholesterol; TG, Triglyceride; WC, waist circumference. ^a,b^ values with different superscripts are statistically significant (within treatment period comparisons of change values between genotypes using linear mixed models, adjusted for age, gender, baseline BMI, amount of consumed oils, change levels of physical activity and change in energy intake). *P* ^1^, comparisons of change values between the treatment oils using linear mixed models, adjusted for age, gender, baseline BMI, amount of consumed oils, change levels of physical activity and change in energy intake. *P* ^2^, comparisons of change values between the genotypes using linear mixed models, adjusted for age, gender, baseline BMI, amount of consumed oils, change levels of physical activity and change in energy intake. *P* ^3^, interaction between TaqIB SNP and treatment oils on the outcomes of interest, adjusted for age, gender, baseline BMI, amount of consumed oils, change levels of physical activity and change in energy intake.

### The effect of dietary oils on blood lipids and apolipoproteins according to the CETP TaqIB genotypes

In patients with type 2 diabetes, B1B1 homozygotes compared with B2 carriers (B1B2 + B2B2) had a significant decrease in apoB levels (-19.05 ± 6.12 mg/dL) and apoB: apoA-1 ratio (-0.12 ± 0.04) and an increase in Lp(a) levels (6.37 ± 2.17 mg/dL) following the intake of sesame-canola oil (Table 1). There was also a significant effect of genotype on change values of apoB (*P* = 0.041) and apoB: apoA-1 ratio (*P* = 0.018) without considering the type of consumed dietary oils (Table 1). Bonferroni adjustment for multiple comparisons showed that the levels of apoB and apoB: apoA-1 ratio have been reduced in B1B1 homozygotes compared with B2 carriers. There was also a significant combined effect of the TaqIB polymorphism and dietary oils on LDL: HDL (*P* = 0.027), TC: HDL (*P* = 0.024), and TG: HDL (*P* = 0.025) (Table 1). These ratios in B1B1 homozygotes tended to decrease following the intake of sesame oil and sesame-canola oil, but tended to increase following the intake of canola oil. No independent or combined effects of dietary oils and genotypes were observed for other outcomes.

In healthy people, there were no significant differences between genotypes regarding the change in outcomes, except a considerable difference for adjusted TC changes between the two genotype groups during the sesame oil treatment period (B1B2: -2.75 ± 3.52 mg/dL; B2B2: 17.06 ± 5.82 mg/dL, *P* < 0.05, Table 2). No independent or combined effects of dietary oils and genotypes were observed for all outcomes (Table 2).

### The effect of dietary oils on glycemic indices according to the CETP TaqIB genotypes

As shown in Table 1, patients with type 2 diabetes carrying B1B1 genotype had a significantly lower insulin (B1B1: -15.07 ± 3.77 mIU/mL vs. B1B2: -4.75 ± 2.20 mIU/mL and B2B2: 0.76 ± 3.77 mIU/mL) and HOMA-IR (B1B1: -1.74 ± 0.45 vs. B2B2: 0.10 ± 0.45) than carriers of the B2 allele in the sesame oil period. No significant differences between genotype groups were observed for other glycemic control markers across all treatment periods. On the other hand, there was a significant effect of genotype on adjusted changes of insulin (*P* = 0.031), HOMA-IR (*P* = 0.024) and QUICKI (*P* = 0.046) without considering the type of consumed dietary oils (Table 1). According to Bonferroni adjustment for multiple comparisons, those with B1B1 genotype compared with B2B2 homozygotes showed a significant reduction in insulin and HOMA-IR and an increase in QUICKI. Glycemic indices did not differ statistically between the *CETP* TaqIB genotypes during all treatment periods in healthy people (*P* > 0.05, Table 2). Also, neither the intervention nor the polymorphism had a significant effect on outcomes changes. We found no combined effect of the TaqIB polymorphism and dietary oils on glycemic control measures in both type 2 diabetic and healthy people (Table 1 and Table 2).

### The effect of dietary oils on liver enzymes according to the CETP TaqIB genotypes

No significant differences between genotype groups were found for liver enzymes in both type 2 diabetes and healthy people (Table 1 and 2), however, during the canola oil period, the levels of AST and ALT were significantly decreased in B1B1 subjects as compared with carriers of the B2 allele only in healthy people (Table 2). There was also a significant combined effect of the TaqIB polymorphism and dietary oils on AST concentrations in healthy people (*P* = 0.019, Table 2). No independent or combined effects of dietary oils and genotypes were observed for other liver enzymes in both type 2 diabetes and healthy people (Table 1 and 2).

## Discussion

Gene-diet interaction is an important emerging field of research that is expected to provide more knowledge about the association between genetic traits and dietary factors in relation to different aspects of health. The number of studies assessing the effects of genetic variations on the metabolic response to dietary interventions is rapidly increasing. To the best of our knowledge, our study is the first clinical trial to investigate the combined effects of the *CETP* TaqIB polymorphism and dietary plant oils on the metabolic response after consuming canola, sesame, and sesame-canola oils in diabetes patients and healthy people. In patients with type 2 diabetes, we found generally a significant genotype effect; such that individuals with B1B1 genotype showed a significant decrease in apoB, apoB: apoA-1, insulin, and HOMA-IR and also an increase in QUICKI compared with B2B2 homozygotes. We also observed a differential effect for this polymorphism depending on the type of dietary plant oil; serum levels of apoB and apoB: apoA-1 ratio favorably decreased in diabetes patients who were B1B1 homozygotes rather than B2 carriers after sesame-canola oil intake, whereas the levels of Lp(a) was adversely increased. Moreover, a considerable genotype-dietary oils combined effects were detected in diabetes patients as LDL: HDL, TC: HDL and TG: HDL ratios tended to decrease following the intake of sesame and sesame-canola oils, but tended to increase following the intake of canola oil in B1B1 homozygotes. Our study was unable to show significantly different responses between the genotype groups as well as gene-dietary oils combined effects in healthy people.

The evidence has shown that CETP activity and the risk of coronary diseases were relative to TaqIB variant; the B2 allele was associated with moderate inhibition of CETP activity, higher HDL-C and apoA-1 levels, lower TG levels and consistently a reduced risk of coronary diseases than did B1B1 individuals.^16,17^ It was also found that the B2 allele tended to decrease the risk of metabolic syndrome.^34^ Moreover, the prevalence of macrovascular complications in diabetes patients such as arteriosclerosis obliterans, coronary heart and cerebrovascular diseases was significantly higher in individuals homozygous for the B1 allele.^35^ Indeed, B1B1 genotype seems to be a risk factor for metabolic disturbances, as we also observed a more frequency of B1 homozygotes in patients with type 2 diabetes than healthy people (18.9% vs. 13.7%), however, no significant association between the TaqIB variant and baseline trait levels was found.

On the other hand, there is evidence that B1B1 genotype is associated with a better response to nutritional interventions compared with the B2 allele.^36^ In a number of researches, B1B1 homozygotes of the *CETP* TaqIB polymorphism displayed a better lipid profile in response to high carbohydrate/low fat dietary intervention,^21^ consuming two green kiwifruit a day alongside a healthy diet,^37^ olive-oil-enriched milk,^22^ and plant stanol ester compared with the B2 allele carriers.^38^ Similarly, in line with a relatively large body of literature, we observed that the dietary oil treatments could be more effective in carriers of the B1B1 homozygous than those with the B2 allele. However, such findings are not necessarily consistent between studies and do not reinforce the idea that this specific polymorphism can affect metabolic responses to interventional therapies.^24,25,39^

CETP activity can be affected by genetic and environmental factors such as dietary intakes, however, the possible roles of genetic variants at the *CETP* locus and mechanisms underlying the effects of these variants on the responsiveness to nutritional interventions in the modulation of lipid metabolism is not clearly established. One possibility is that the combined effects of dietary factors and *CETP* polymorphisms on metabolic responses are modulated through their effects on CETP activity. The TaqIB mutation is located at the position 277 in the first intron of the *CETP* gene and so it is very unlikely to be a functional mutation; however, this SNP is in linkage disequilibrium with a mutation in the *CETP* promoter, which is known to have functional effects.^40,41^ On the other hand, it has been proposed that dietary fats can play roles in modulating CETP activity, such that in contrast to SFAs, the intake of monounsaturated and polyunsaturated fats has been associated with decreased activity of CETP.^42-45^

There is also insufficient evidence to assess whether glycemic responsiveness is affected by variation in the *CETP* gene. Lopez-Rios et al^18^ reported an increased levels of insulin and HOMA in individual homozygotes for the B1 allele rather than subjects carrying at least one B2 allele that may suggest the effect of the TaqIB polymorphism on parameters of insulin resistance. Our findings also revealed a modulatory effect of the TaqIB variant on insulin, HOMA-IR, and QUICKI in response to dietary oil treatments only in diabetes patients.

The strength of our study was in its design as a randomized, cross-over study and including a relatively large sample size which minimizes the inter-individual variations and differences in diet responsiveness. The analysis was also conducted in subjects with and without diabetes separately, making our findings attributable to both populations. Moreover, in our study, the regular oils used in households were fully replaced with dietary oil treatments which is easier to implement in real life. In other words, despite most of the clinical trials, we did not use specific amounts of oils, and participants were free to consume dietary oils ad libitum. However, it must be noted that the studies assessing gene-diet interactions like our study face some limitations. Taking into account that the metabolic responses to dietary changes are under polygenic control and each gene probably contribute to a relatively small effect, in such studies which commonly focused on single gene effects only, the possibility of statistically significant gene-diet interactions is reduced. Moreover, most of such researches were not specifically designed to assess gene-diet interactions and were retrospective analysis of collected data. Indeed, participants were not selected based on their genotypes and this approach may result in small numbers of individuals in each genotype group especially for the rare allele. On the other hand, we did not measure CETP activity directly and the effect of the TaqIB variant on concentration and activity of CETP could not be assessed; therefore it is difficult to discuss the mechanisms underlying the modification effects of this polymorphism. Another limitation in our study was that we could not measure different subclasses of HDL particles, since HDL_3_ subspecies may be a more sensitive marker in relation to the effects of CETP activity on HDL metabolism.^46^

## Conclusion

In conclusion, our findings suggest that genetic variation at the *CETP* gene may contribute to the heterogeneity in responsiveness of some metabolic traits to dietary oil treatments in patients with type 2 diabetes. However, no significant modulatory effect of the *CETP* TaqIB polymorphism on metabolic traits in response to plant oils was found in healthy people. Taken together, the intake of sesame-canola and canola oils showed more favorable effects in diabetes patients with B1B1 genotype. The evidence of gene-diet interactions is limited and there is a need for further precise investigations on the effects of polymorphisms in multiple genes simultaneously, and not only in single genes to increase our knowledge of the mechanisms underlying the modulation of genetic variants and dietary factors on metabolic metabolism.

## Competing interests

The authors declare that Datis Corporation had no role in the design and conduct of the study; collection and management of the data, analysis and interpretation of the data; and also manuscript preparation.

## Acknowledgements

The authors acknowledge all of the participants of the study as well as Shahid Sadoughi University of Medical Sciences (http://www.ssu.ac.ir) and Neshatavar food industry company (Datis Corporation, http://www.neshatavar.com/?l=EN) that jointly financially supported this project. The authors are also thankful for scientific support from the research council of Nutrition and Food Security Research Center and Diabetes Research Center of Shahid Sadoughi University of Medical Sciences, Yazd, Iran.

## Ethical approval

Ethically approved by the ethics committee of Shahid Sadoughi University of Medical Sciences, Yazd, Iran (IR.SSU.SPH.REC.1397.139).

## Funding

The study was jointly funded by Shahid Sadoughi University of Medical Sciences (http://www.ssu.ac.ir: grant number: 5799) and Neshatavar food industry company (Datis Corporation, http://www.neshatavar.com/?l=EN: grant number: 6462).

## Supplementary materials


Supplementary file 1 contains Tables S1 and S2.
