# Supplementary material for: The combined effects of cholesteryl ester transfer protein (CETP) TaqIB gene polymorphism and canola, sesame and sesame-canola oils consumption on metabolic response in patients with diabetes and healthy people
Source: J Cardiovasc Thorac Res. 2020 Aug 2;12(3):185–94. doi: 10.34172/jcvtr.2020.32 (PMC7581849; doi:10.34172/jcvtr.2020.32)
Supplement: Supplementary file 1 — contains Tables S1 and S2. [file jcvtr-12-185-s001.pdf]

Supplementary file 1

Table S1. Baseline characteristics of type 2 diabetic and healthy people according to the *CETP* TaqIB genotypes[1]

	Type 2 diabetes			*P*	Healthy			*P*
	**B1B1**	**B1B2**	**B2B2**		**B1B1**	**B1B2**	**B2B2**	
**Number, F**	18, 11	58, 28	19, 10	0.632	10, 5	47, 27	16, 9	0.911
**Age, y**	48.32 (1.63)	49.24 (0.90)	49.80 (1.58)	0.805	45.16 (2.58)	47.77 (1.19)	47.88 (2.04)	0.641
**Weight, kg**	77.14 (2.74)	76.68 (1.52)	76.59 (2.66)	0.987	72.39 (3.84)	76.61 (1.76)	72.74 (3.02)	0.406
**BMI, kg/m^2**	29.46 (0.87)	28.99 (0.48)	28.24 (0.85)	0.600	27.45 (1.45)	28.90 (0.66)	26.67 (1.13)	0.213
**WC, cm**	101.14 (2.12)	101.12 (1.17)	100.59 (2.06)	0.974	96.68 (3.37)	100.27 (1.54)	97.02 (2.65)	0.433
**Visceral fat, %**	10.78 (0.71)	10.68 (0.39)	10.58 (0.69)	0.979	9.21 (0.95)	9.66 (0.44)	8.24 (0.75)	0.276
**Body fat, %**	34.91 (1.31)	33.87 (0.73)	32.02 (1.27)	0.275	33.48 (1.78)	34.67 (0.83)	31.44 (1.39)	0.145
**Muscle mass, %**	29.19 (0.58)	29.77 (0.32)	30.13 (0.57)	0.518	29.49 (0.78)	29.13 (0.36)	30.25 (0.61)	0.304
**SBP, mmHg**	9.88 (0.31)	10.50 (0.17)	10.27 (0.30)	0.225	10.34 (1.55)	10.16 (0.71)	12.67 (1.22)	0.207
**DBP, mmHg**	7.02 (0.26)	7.49 (0.14)	7.26 (0.25)	0.278	7.31 (0.34)	7.23 (0.15)	7.30 (0.27)	0.958
**TC, mg/dl**	159.66 (7.37)	161.40 (4.09)	162.28 (7.15)	0.966	177.19 (10.97)	182.07 (5.03)	181.79 (8.61)	0.920
**HDL-C, mg/dl**	36.24 (2.46)	38.44 (1.36)	39.09 (2.38)	0.672	42.01 (3.29)	41.37 (1.50)	44.24 (2.58)	0.635
**LDL-C, mg/dl**	80.77 (4.36)	80.97 (2.42)	79.87 (4.22)	0.975	90.63 (7.12)	93.70 (3.26)	93.29 (5.59)	0.926
**TG, mg/dl**	161.70 (18.04)	152.69 (10.01)	159.41 (17.49)	0.885	122.52 (18.71)	139.93 (8.58)	140.90 (14.70)	0.682
**Lp(a), mg/dl**	22.61 (6.01)	24.01 (3.20)	19.06 (5.49)	0.739	19.62 (7.14)	24.96 (3.10)	29.67 (5.49)	0.533
**LDL: HDL**	2.49 (0.33)	2.46 (0.18)	2.23 (0.32)	0.808	2.37 (0.52)	2.63 (0.24)	2.15 (0.41)	0.596
**TC: HDL**	4.98 (0.69)	4.93 (0.38)	4.52 (0.67)	0.851	4.60 (1.15)	5.16 (0.52)	4.19 (0.90)	0.630
**TG: HDL**	5.63 (1.48)	5.52 (0.82)	4.76 (1.44)	0.885	3.59 (2.61)	5.01 (1.20)	3.32 (2.05)	0.733
**ApoB, mg/dl**	107.46 (7.98)	93.03 (4.43)	88.04 (7.73)	0.186	102.73 (10.88)	105.38 (4.99)	98.61 (8.55)	0.790
**ApoA-1, mg/dl**	149.78 (5.95)	150.65 (3.30)	158.73 (5.77)	0.438	149.30 (8.22)	159.76 (3.77)	167.29 (6.46)	0.235
**ApoB: ApoA-1**	0.73 (0.06)	0.63 (0.03)	0.57 (0.06)	0.168	0.69 (0.07)	0.67 (0.03)	0.59 (0.06)	0.448
**FBG, mg/dl**	112.14 (6.63)	115.04 (3.68)	120.34 (6.42)	0.658	87.32 (3.84)	88.27 (1.76)	85.10 (3.01)	0.664

**Insulin, mIU/ml**	31.08 (5.07)	29.20 (2.68)	23.60 (4.89)	0.518	22.71 (3.92)	24.95 (1.92)	22.38 (3.18)	0.738
**HOMA-IR**	3.94 (0.60)	3.76 (0.32)	2.96 (0.56)	0.407	2.80 (0.45)	3.08 (0.22)	2.73 (0.36)	0.663
**QUICKI**	0.29 (0.006)	0.29 (0.003)	0.29 (0.006)	0.942	0.30 (0.007)	0.30 (0.003)	0.31 (0.005)	0.542
**ALP, U/L**	173.94 (10.94)	182.47 (6.07)	206.62 (10.61)	0.075	182.52 (13.56)	186.68 (6.22)	173.22 (10.66)	0.554
**GGT, U/L**	29.11 (3.38)	29.43 (1.88)	27.98 (3.28)	0.929	23.76 (4.06)	25.72 (1.86)	17.90 (3.19)	0.114
**AST, U/L**	22.40 (2.87)	23.70 (1.59)	25.08 (2.78)	0.799	21.41 (3.12)	24.99 (1.43)	19.07 (2.45)	0.103
**ALT, U/L**	24.64 (3.86)	26.97 (2.14)	25.66 (3.74)	0.857	18.73 (4.22)	22.75 (1.93)	16.35 (3.32)	0.225

[1]All data are presented as mean (standard error), age and gender adjusted values. The comparisons were done using one-way analysis of variance (ANOVA). ApoA-1, apolipoprotein A-1; ApoB, apolipoprotein B; ALP, alkaline phosphatase; AST, aspartate aminotransferase; ALT, alanine aminotransferase, BMI, body mass index; DBP, diastolic blood pressure; FBG, fasting blood glucose; GGT, gamma-glutamyltransferase; HDL-C, High-density lipoprotein cholesterol; HOMA-IR, homeostasis model assessment for insulin resistance; LDL-C, Low-density lipoprotein cholesterol; Lp(a), lipoprotein a; QUICKI, quantitative insulin sensitivity check index; SBP, Systolic blood pressure; TC, total cholesterol; TG, Triglyceride; WC, waist circumference.

Table S2. Total energy, nutrient intakes and physical activity of patients with type 2 diabetes and healthy people according to each treatment period[1]

	**Type 2 diabetes (n = 95)**			***P***	**Healthy (n = 73)**			***P***
	**Sesame oil**	**Sesame-Canola oil**	**Canola oil**		**Sesame oil**	**Sesame-Canola oil**	**Canola oil**	
**Energy (Kcal/day)**	1764.42 (37.61)	1805.77 (37.65)	1768.20 (37.70)	0.298	1795.55 (47.27)	1843.35 (47.49)	1817.51 (47.33)	0.341
**Carbohydrate (%E)**	58.95 (0.60)	59.55 (0.60)	59.04 (0.60)	0.635	58.91 (0.62)	60.44 (0.62)	59.50 (0.62)	0.118
**Protein (%E)**	15.48 (0.20)	15.38 (0.20)	15.49 (0.20)	0.856	14.89 (0.24)	14.98 (0.24)	14.83 (0.24)	0.856
**Total Fat (%E)**	27.21 (0.50)	26.63 (0.50)	27.18 (0.49)	0.488	27.78 (0.54)	26.27 (0.54)	27.28 (0.54)	0.054
**SFA (%E)**	7.93 (0.17)	7.73 (0.17)	7.80 (0.17)	0.638	8.27 (0.22)[a]	7.66 (0.22)[b]	7.80 (0.22)	0.044
**MUFA (%E)**	8.46 (0.20)[a]	9.06 (0.20)[b]	9.75 (0.20)[c]	<0.001	8.85 (0.22)[a]	8.91 (0.22)[a]	9.63 (0.22)[b]	0.002
**PUFA (%E)**	6.25 (0.21)[a]	5.34 (0.20)[b]	5.60 (0.20)[b]	0.001	6.15 (0.23)[a]	5.18 (0.23)[b]	5.31 (0.22)[b]	0.004
**PA (MET-min/day)**	2182.69 (26.56)	2144.98 (26.58)	2182.88 (26.70)	0.190	2185.52±30.60	2187.71 (31.24)	2150.93 (30.54)	0.238

[1]All data are presented as mean (standard error). The comparisons were done using linear mixed models ([a,b,c] values with different superscripts are statistically significant). SFA, saturated fat; MUFA, mono unsaturated fatty acid; PUFA, poly unsaturated fatty acid; PA, physical activity; MET, metabolic equivalent.
